# Genome-wide analysis of circular RNA-mediated ceRNA regulation in porcine skeletal muscle development

**DOI:** 10.1186/s12864-023-09284-7

**Published:** 2023-04-12

**Authors:** Jiale Yun, Xiaoyu Huang, Chang Liu, Mingyue Shi, Wenxia Li, Jin Niu, Chunbo Cai, Yang Yang, Pengfei Gao, Xiaohong Guo, Bugao Li, Chang Lu, Guoqing Cao

**Affiliations:** College of Animal Science, Shanxi Agricultural University, Taigu, 030801 China

**Keywords:** Pig, Skeletal muscle, RNA-seq, circRNA, ceRNA

## Abstract

**Background:**

As a diverse and abundant class of endogenous RNAs, circular RNAs (circRNAs) participate in various biological processes including cell proliferation and apoptosis. Nevertheless, few researchers have investigated the role of circRNAs in muscle development in cultivated pigs.

**Results:**

In this study, we used RNA-seq to construct circRNA expression profiles in skeletal muscle of Jinfen White pigs at the age of 1, 90, and 180 days. Among the 16,990 identified circRNAs, 584 circRNAs were differentially expressed. Moreover, the enrichment analysis of DE circRNA host genes showed that they were mainly involved in muscle contraction, muscle organ development and muscle system processes, as well as AMPK and cAMP-related signal pathways. We also constructed a circRNA–miRNA–mRNA co-expression network to find key circRNAs which many involved in the regulation of porcine skeletal muscle development through the competitive endogenous RNA (ceRNA) mechanism. It is noteworthy that circ_0018595/miR-1343/*PGM*1 axis may play a regulatory role in the development of porcine skeletal muscle.

**Conclusions:**

This study identified the circRNAs and present the circRNA expression profile in the development of pigs, revealed that DE circRNA host genes participate in different cell fates and enriched the porcine ceRNA network. Thus, this work will become a valuable resource for further in-depth study of the regulatory mechanism of circRNA in the development of porcine skeletal muscle.

**Supplementary Information:**

The online version contains supplementary material available at 10.1186/s12864-023-09284-7.

## Background

Skeletal muscle is the main tissue of the animal body, and it has important functions in exercise, heat production, maintenance of body shape, storage of protein, and protection of organs [1, 2]. Muscle fiber is the basic functional unit of skeletal muscle, and its quantity remains unchanged after the birth of mammals. The growth and development of skeletal muscle is achieved through the hypertrophy of existing muscle fiber [3]. In the process of myogenesis, skeletal muscle satellite cells gradually form mature muscle tissue after undergoing myoblasts, multinucleated myotubes, muscle fibers and other stages, and play an indispensable role in the growth of skeletal muscle [4, 5]. Regulation of the activity of myogenic stem cells involves many transcription factors, such as myoblast determination protein 1 (*MyoD1*), myogenin (*MyoG*), myogenic factor 5 (*Myf5*), and myogenic factor 6 (*Myf6*), as well as other myogenesis-related regulatory transcription factors, including the paired box 3 (PAX3), paired box 7 (*PAX7*), and myocyte enhancer factor 2 (*MEF2*) families [6–9]. Among these, *MyoD1* and *Myf5* are involved in the first stage of skeletal muscle development, which promoting the proliferation and differentiation of myogenic progenitor cells into myoblasts, while *MyoG* determines the generation of myotubes, and *Myf6* is involved in cell differentiation and cell fate [10–13]. Furthermore, the expression of *MyoG* is regulated by *MRF4* and *MyoD* [14].

In addition to the above roles of transcription factors, a growing number of studies have established that non-coding RNAs (ncRNAs) are also involved in the regulation of myogenesis. These include microRNAs (miRNAs), long non-coding RNAs (lncRNAs), and circular RNAs (circRNAs), whose regulatory roles in the growth of skeletal muscle are gradually being revealed [15–17]. With the development of high-throughput sequencing technology, circRNA has received increasing attention in recent years. As the most recently emerging class of ncRNA, circRNA is essentially a closed RNA transcript that is produced by reverse splicing of precursor mRNA (pre-mRNA). The 3′ and 5′ ends are covalently linked, and they differ from their linear counterparts in the absence of a 5′ cap and polyadenylated [poly(A)] tail [18]. Moreover, circRNAs have been identified in most species, and are generally expressed at low levels, but partly more abundantly than their host genes (also referred to as source genes) [19–21]. Compared with lncRNAs, circRNAs display highly conserved expression patterns and transcript sequences among species, and they are widely expressed in various tissues and organs [22, 23]. Much current research on circRNAs focuses on aspects of cancer, including cell proliferation, apoptosis, invasion, and chemoresistance [24–27].

The research on circRNA in skeletal muscle mainly focuses on the proliferation and differentiation of myoblasts. CircRBFOX2 and circFGFR2 can regulate the development of chicken myoblasts, which can sponge and negatively regulate miR-206 and miR-133a-5p respectively [28, 29]. CircFAM188B encoded a novel protein named circFAM188B-103aa that promoted the proliferation of chicken skeletal muscle satellite cells but suppressed differentiation [30]. CircFUT10 sponge miR-133a leaded to inhibition of cell proliferation and ultimately enhanced myoblastic differentiation [31]. At present, the research on circRNA regulation of porcine skeletal muscle growth mainly depends on the in-depth mining of sequencing data. Sun constructed a pig skeletal muscle injury model and screened out the differentially expressed circRNA circSCDE1. Because it is highly conservative in pigs and mice, C2C12 was used to verify the specific mechanism of circCSDE1 regulating the proliferation and differentiation of myoblasts [32]. Ma identified and predicted circRNA in Duroc pigs with different daily weight gain [33]. Yan screened the highly conservative circFGFR2, and explained that circFGFR2 regulates myogenesis and skeletal muscle regeneration through feedback loop on C2C12 [34]. Jinfen White pigs are cultivated from four parental breeds, which have the advantages of good meat quality, strong resistance to stress, and rapid growth [35–37]. However, in Jinfen White pigs, the characteristic and mechanism of regulation by circRNAs at different growth stages are still unclear. Thus, further exploration is needed.

Therefore, in this study, RNA-seq data in the longissimus dorsi muscle of Jinfen White pigs were sampled at three growth stages (9 samples: 1 d, 90 d and 180 d). We investigated the feature, expression and potential function of identified circRNAs in Jinfen White pigs and constructed a ceRNA network to find the key circRNAs in the development of pigs. This will advance our knowledge of the feature and predicted function of the circRNAs during the developmental stages of pigs.

## Results

### Overview of sequencing data

To understand the expression characteristics of circRNA in porcine muscle at different developmental stages, we performed ribosomal RNA-depleted RNA-seq on muscle sampled at three time points. Firstly, we constructed nine non-coding RNA libraries named JFW_1d_1, JFW_1d_2, JFW_1d_3, JFW_90d_1, JFW_90d_2, JFW_90d_3, JFW_180d_1, JFW_180d_2, and JFW_180d_3. Secondly, we purified and sequenced the RNA using the Illumina paired-end RNA-seq method. For these libraries, an average of 100,961,956 raw reads were obtained from the platform, and after filtering, a final dataset of 100,294,750 clean reads was obtained (Table 1). The JFW_180d_3 group contained the greatest number of reads (106,567,824), and the JFW_1d_1 group had the least (91,942,766). The Q30 quality threshold (Phred quality score > 30) was met for 95.2% of the data. Taken together, these results confirmed that the sample and sequencing data generated in this study were of good quality and were reliable for subsequent bioinformatic analysis.

**Table 1:** Overview of RNA-seq results for each sample

Sample name	Raw reads	Clean reads	Raw_bases (G)	Clean_bases (G)	Error rate (%)	Q20 (%)	Q30 (%)
JFW_1d_1	92,655,706	91,942,766	13.9	13.79	0.02	98.62	95.72
JFW_1d_2	107,050,232	106,444,968	16.06	15.97	0.02	98.64	95.78
JFW_1d_3	104,392,040	103,814,406	15.66	15.57	0.02	98.58	95.45
JFW_90d_1	96,202,328	95,550,798	14.43	14.33	0.03	98.08	94.2
JFW_90d_2	106,311,518	105,563,842	15.95	15.83	0.02	98.56	95.75
JFW_90d_3	95,622,436	94,984,938	14.34	14.25	0.02	98.67	95.89
JFW_180d_1	94,853,354	94,375,978	14.23	14.16	0.03	97.81	93.78
JFW_180d_2	104,189,042	103,407,232	15.63	15.51	0.02	98.59	95.54
JFW_180d_3	107,380,946	106,567,824	16.11	15.99	0.02	98.40	95.04
average	100,961,956	100,294,750	15.15	15.04	0.02	98.44	95.24

### Characterization of circRNAs in porcine skeletal muscle

**Fig. 1:**
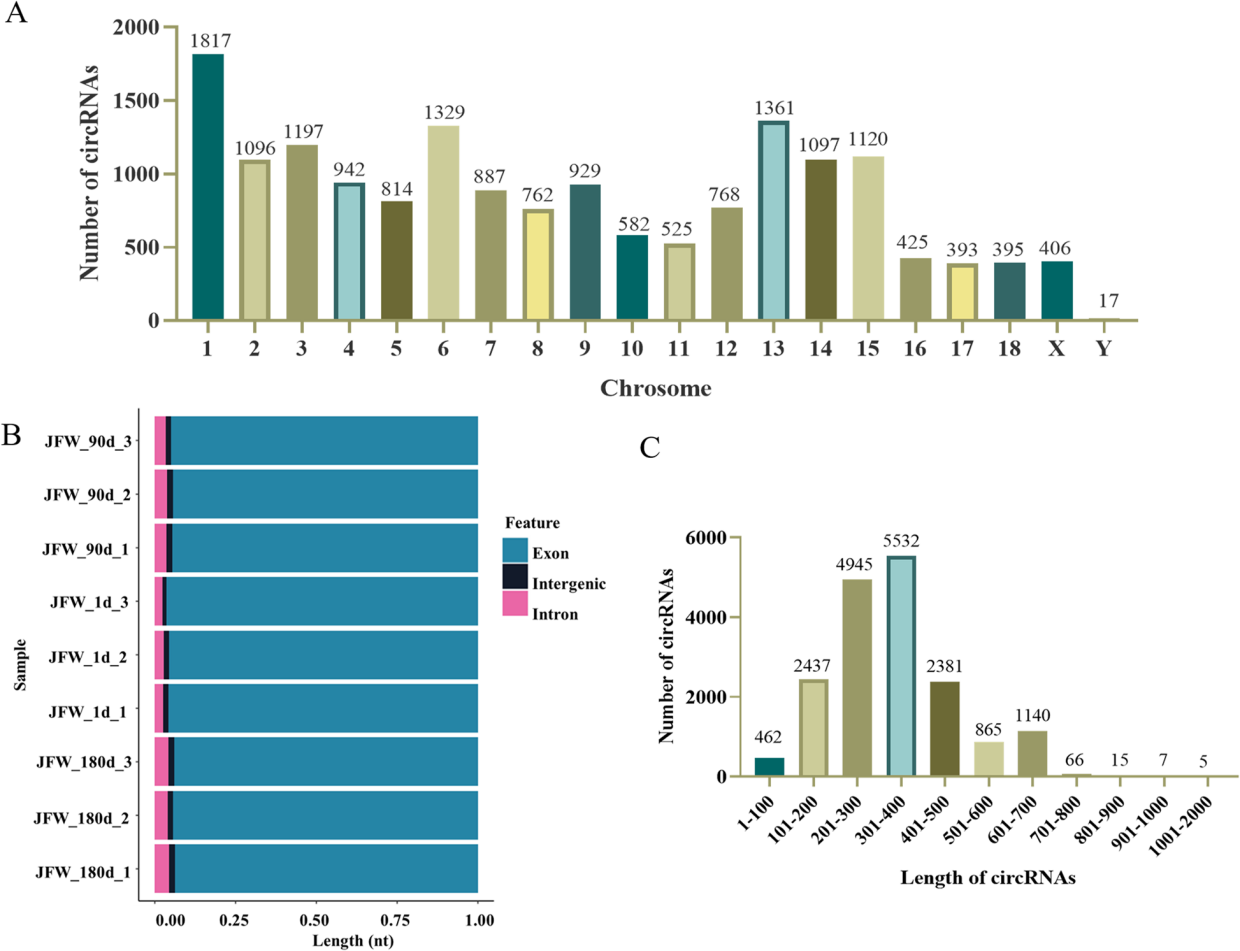
Features of identified circRNAs. **A** Chromosomal distribution of circRNAs from all samples. **B** Source distribution of circRNAs for each sample. **C** Length distribution of circRNAs for all samples

After mapping the clean data to the pig genome (Sus scrofa11.1) using the find_circ and CIRI software, the intersection selected by the two software is used as the circRNAs for final identification. We identified a total of 16,990 highly credible circRNAs (expressed in at least three or more samples) from the nine libraries, which were transcribed from 4775 source genes (summarized in more detail in Supplementary Table S1). In addition, we mapped the chromosomal distribution of these circRNAs, finding them to be transcribed from all chromosomes but with an uneven distribution (Fig. 1A). In agreement with other earlier work [38], we observed that the highest number of circRNAs originated from chromosomes 1, 6, and 13 in this study (Fig. 1A). Furthermore, since circRNAs are primarily derived from annotated exons [39], we also found that > 90% identified circRNAs in this study contained exon sequences (Fig. 1B). The length distribution of the predicted circRNAs showed that most of them were less than 1,000 nt in length (Fig. 1C), which was also consistent with previous reports [40].

### Differential expression of circRNAs

**Fig. 2:**
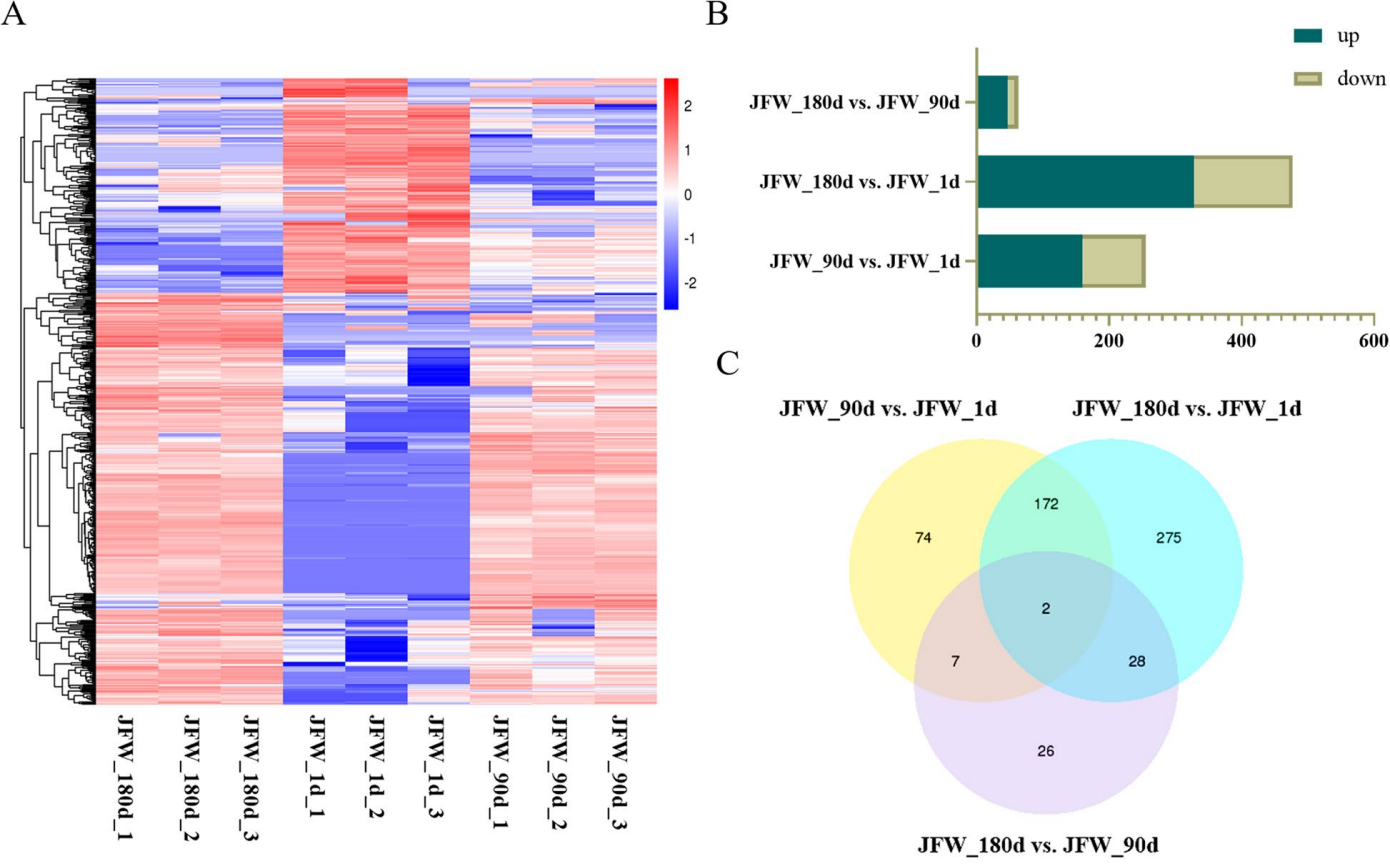
Expression and number of the DE circRNAs at different developmental stages. **A** Expression heatmap of DE circRNAs from samples at three different growth stages, with rows showing circRNAs and columns showing tissues. **B** The number of differentially expressed circRNAs in each comparison including JFW_90d vs. JFW_1d, JFW_180d vs. JFW_1d, and JFW_180d vs. JFW_90d groups. **C** Venn diagram showing the number of shared DE circRNAs among the three pairwise comparisons

To better understand the differences in circRNA expression patterns in pig muscle at 1, 90, and 180 days, we used DESeq software to identify DE circRNAs between sample pairs (i.e., JFW_90d vs. JFW_1d, JFW_180d vs. JFW_1d, and JFW_180d vs. JFW_90d). We also performed hierarchical clustering analysis of the DE circRNAs. This revealed distinct differential expression patterns for the identified circRNAs between the three time points in muscle, with JFW_1d showing significant differences compared to the other two groups (Fig. 2A). In total, 584 DE circRNAs were detected using the above pairwise comparisons. The number of differentially expressed genes was 477, 63, and 255 for the JFW_180d vs. JFW_1d, JFW_180d vs. JFW_90d, and JFW_90d vs. JFW_1d comparisons, respectively (Fig. 2B). We further constructed a Venn diagram of the identified DE circRNAs in the three pairwise comparisons to find the shared circRNAs (Fig. 2C). The expression level of two kinds of circRNA (circ_0009188 and circ_0018581) increased significantly with age in the three growth stages of Jinfen White pigs. One of these, circ_0009188, is generated by circularization of two exons of tubulin tyrosine ligase-like 7 (*TTLL7*) and is 311 nt in length. The other, circ_0018581, arises through circularization of two exons of glutamate decarboxylase-like protein 1 (*GADL1*), and has a length of 608 nt.

### Validation of DE circRNAs at different developmental stages of muscle

**Fig. 3:**
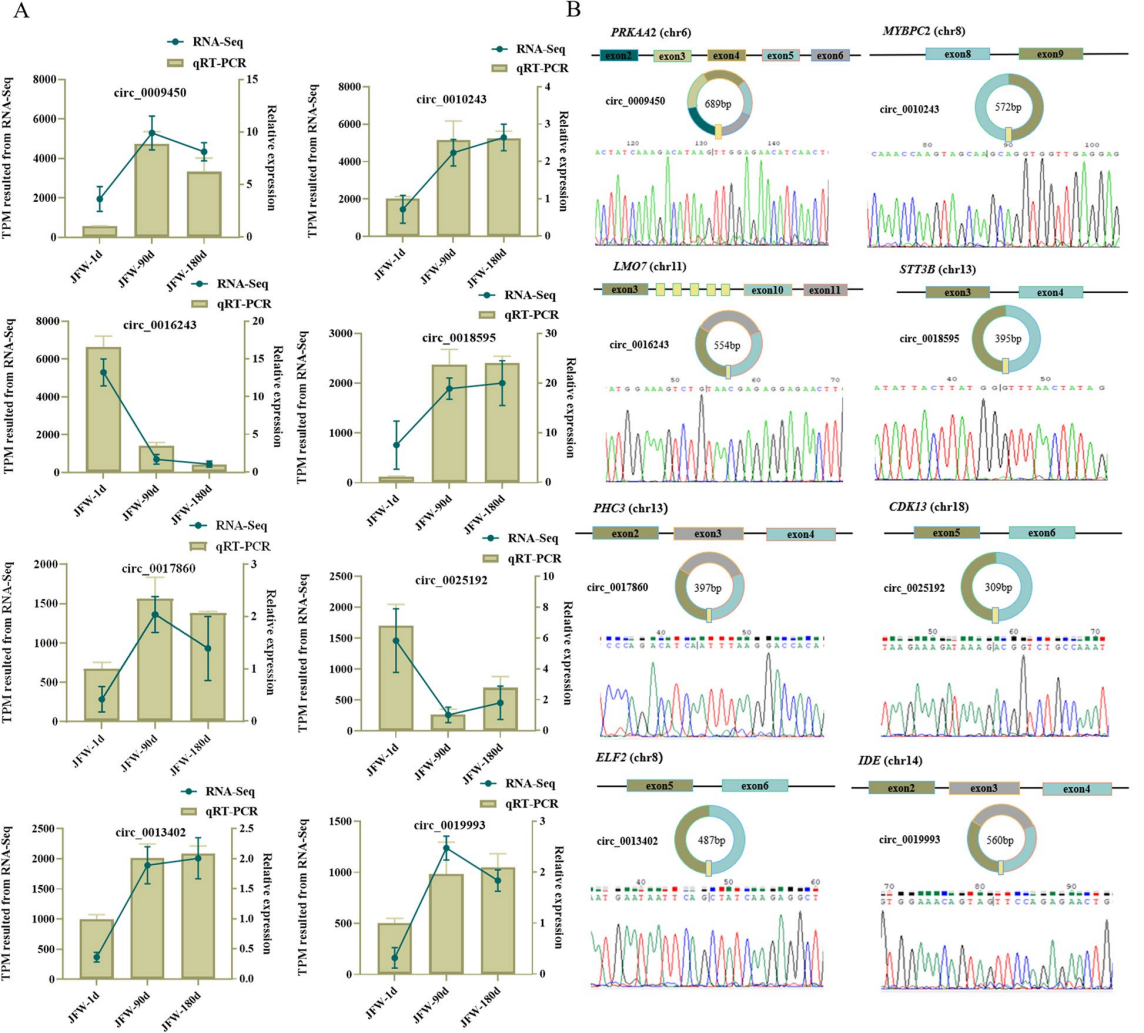
Validation of a subset of the putative circRNAs. **A** Validation of differential expressed circRNAs using qRT-PCR. **B** Representative examples of PCR products purified and sequenced to confirm circRNA junction sequences

To validate the reliability of temporal circRNA expression profiles derived from RNA-seq data, divergent primers were designed for eight randomly selected DE circRNAs, and the expression levels of which were quantified by qRT-PCR (Fig. 3A). The results were consistent with the expression patterns of these circRNAs obtained through RNA-seq data. In addition, the expected size PCR products were obtained by amplification using cDNA as the PCR template. The post-splice sites were verified by Sanger sequencing (Fig. 3B). These results suggested that the identified circRNAs in this study are credible.

### Functional enrichment analysis of DE circRNA host genes

**Fig. 4:**
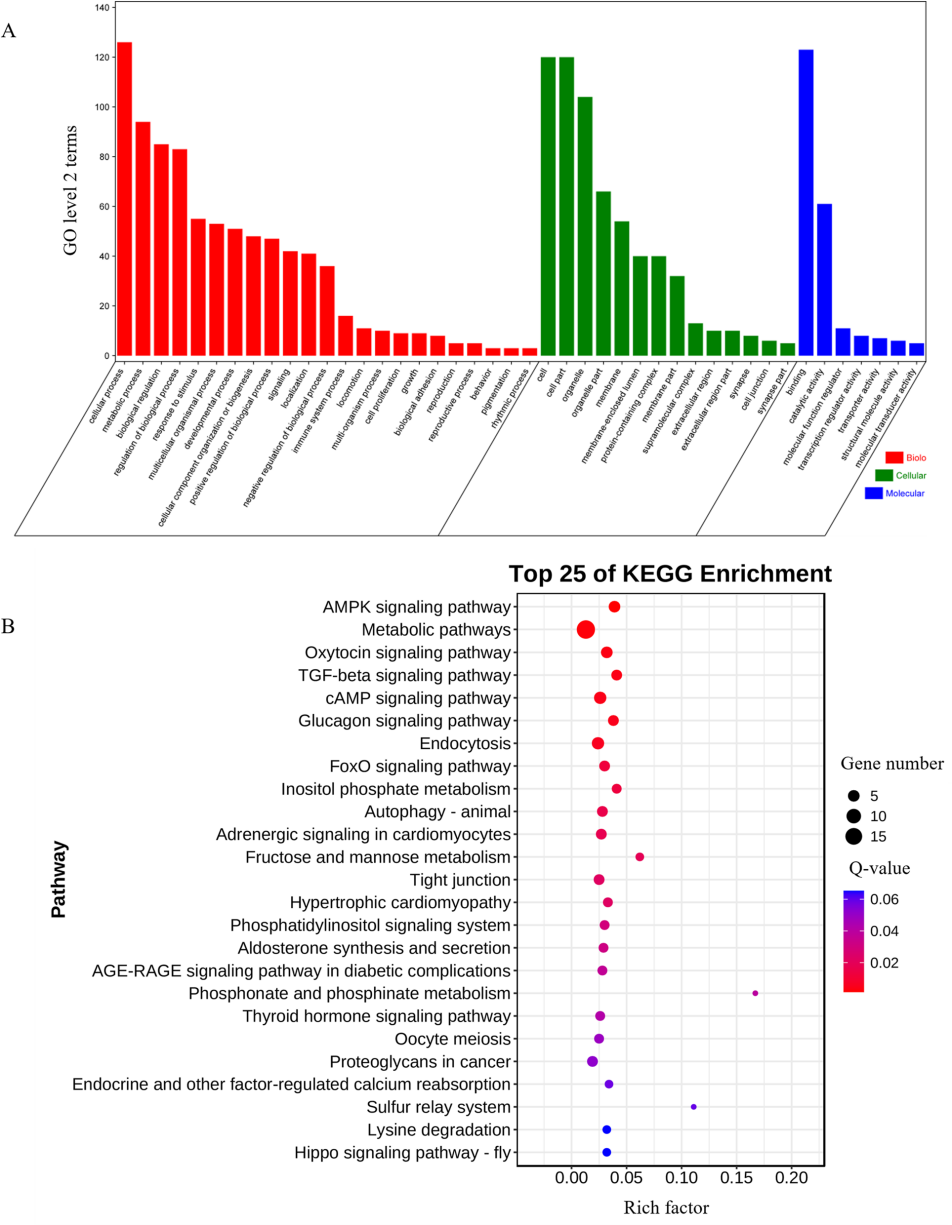
Functional analysis of the DE circRNAs-host genes. **A** Top 25 GO terms enriched for DE circRNA host genes from the three groups of pairwise comparisons. The y-axis presents the number of DE circRNA host genes in a category, while the x-axis shows the specific GO term. The red histogram represents biological processes, green histogram represents cellular components, and the blue histogram represents molecular functions. **B** Top 25 KEGG pathways enriched for DE circRNAs host genes from the three pairwise comparisons groups. The x-axis represents the rich factor (number of DE circRNAs enriched in the pathway / number of annotated pathway genes), while the y-axis shows the specific pathways

Previous studies have shown that circRNAs could regulate host gene transcription by competing with linear pre-mRNA splicing [41, 42]. To explore the potential functions of the identified 584 DE circRNAs in the development of skeletal muscle, we performed GO and KEGG pathway enrichment analyses of their host genes [43]. Notably, we found that these host genes mainly have known roles in muscle biology, including muscle contraction, muscle organ development, muscle system processes, and muscle structure development (Fig. 4A). The host genes *CHD2*, *SMAD3*, *BMPR1A*, *HOMER1*, *DMD*, and *MYL2* are involved in muscle organ development; *HOMER1*, *DMD*, and *ATP11A* are involved in myotube differentiation; and *NFATC1*, *BMPR1A*, and *PDCD4* are involved in the negative regulation of muscle cell differentiation. The KEGG pathway enrichment analysis showed that the DE circRNA host genes were enriched in top 25 pathways, including skeletal muscle fiber-related signaling pathways, and the cAMP and AMPK signaling pathways and so on (Fig. 4B). *PDE4B*, *NFATC1*, *PLCE1*, *ATP2B4, PPP1R12A*, and *CAMK2B* were enriched in the cAMP signaling pathway, and *EEF2K*, *FBP2*, *PPP2CB*, *PFKFB1*, and *PRKAA*2 were enriched in AMPK signal pathway.

### Construction of a potential circRNA-miRNA-mRNA regulatory network

**Fig. 5:**
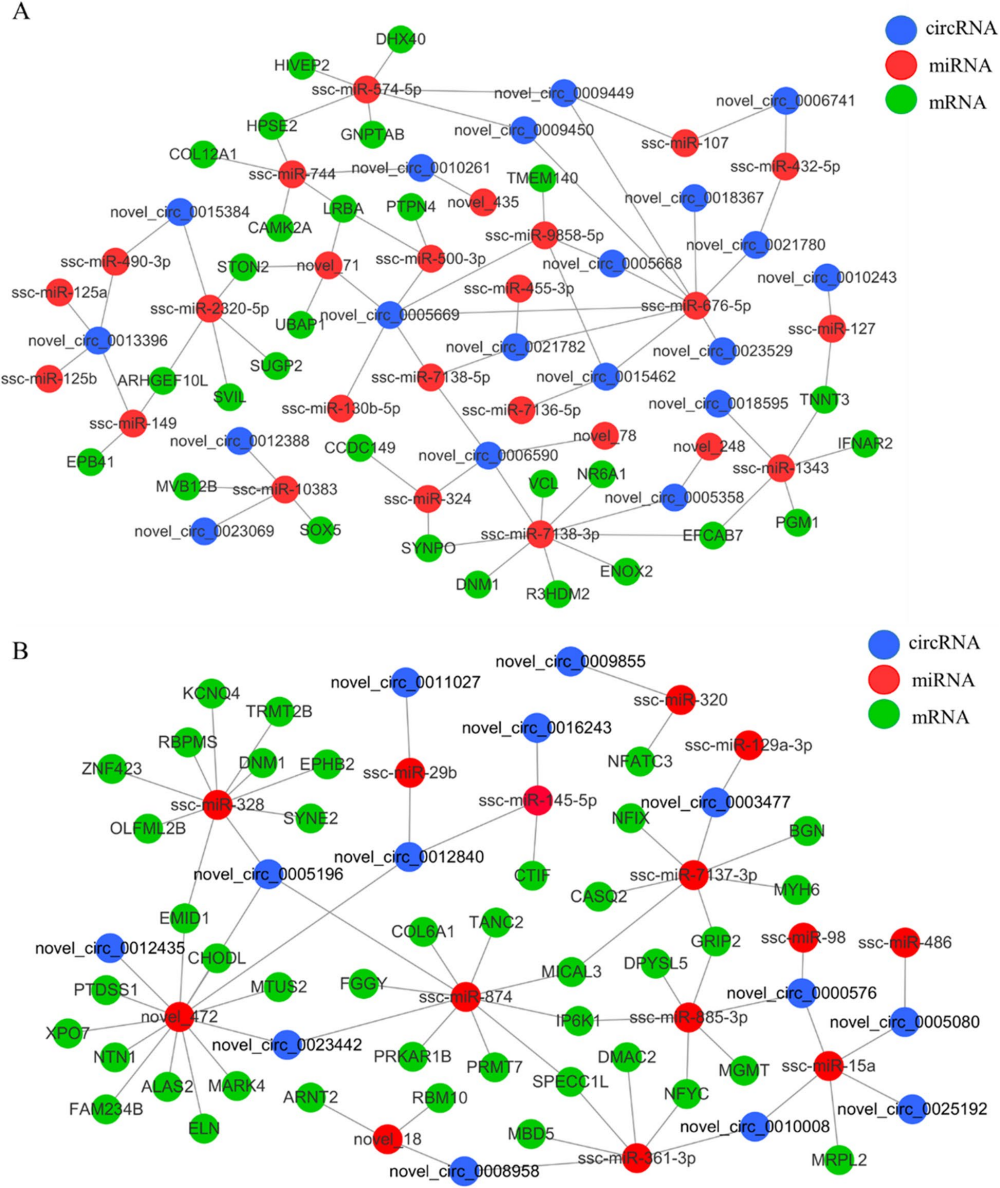
The parts of constructed circRNA–miRNA–mRNA (ceRNA) network. The ceRNA networks showing the top 20 DE circRNAs with the most significant upregulation (**A**) or downregulation (**B**) across the three muscle developmental stages in Jinfen White pigs

CircRNAs often act as miRNA molecular sponges to indirectly regulate the expression of miRNA target genes, thereby meaning that they participate in the related biological processes [44]. Therefore, we integrated miRNA and mRNA library data (The sequencing results obtained at the same time, the accession number is consistent with the circRNA) with the results from the present study to constructed a ceRNA network and identified key circRNAs associated with pork quality and regulation of cell proliferation and differentiation. This ceRNA network contained 229 circRNAs, 55 miRNAs and 143 mRNAs (Supplementary Table S2). Because the ceRNA network is too large, we notice the top 20 DE circRNAs with the most obvious up- or down-regulation profiles to present two ceRNA network maps (Fig. 5). In ceRNA network, circ_0018595 is predicted to function as a sponge for ssc-miR-1343, which binds to the mRNAs transcribed from the *PGM1*, *TNNT3*, and *IFNAR2* genes (Fig. 5A).

To explore the functional significance of mRNAs in the circRNA–miRNA–mRNA regulatory network, we performed functional enrichment analysis on 143 targeted mRNAs. As a result the GO enrichment analysis showed that these target mRNAs were significantly enriched in pyruvate metabolism, hexose metabolism, striated muscle contraction, glycolysis process, muscle system process, and muscle contraction. More specifically, *PGAM2*, *ALDOA*, *ACTN3*, *PKM*, and *GPI* are involved in the process of glycolysis, and *ACTN3*, *PI16*, *WDR1* are involved in striated muscle development. It is worth noting that *ACTN3* is also involved in the positive regulation of fast-twitch skeletal muscle fiber contraction, skeletal muscle fiber development, skeletal muscle tissue growth, and other important processes (Supplementary Figure S1A). Furthermore, KEGG enrichment analysis showed that the same target mRNAs were mainly enriched in carbon metabolism, glycolysis/gluconeogenesis, and regulation of the actin cytoskeleton. Specifically, *PGAM2*, *ALDOA*, *GPI*, and *EFCAB7* are involved in glycolysis and gluconeogenesis, *HOMER3* and *PDPK1* are involved in the FOXO signaling pathway, and *PDPK1*, *ITGA5*, and *ITGB4* are associated with the PI3K-AKT signaling pathway (Supplementary Figure S1B). These results suggested that these circRNAs in the ceRNA network may play important role in the muscle development.

### Experimental validation of circ_0018595

**Fig. 6:**
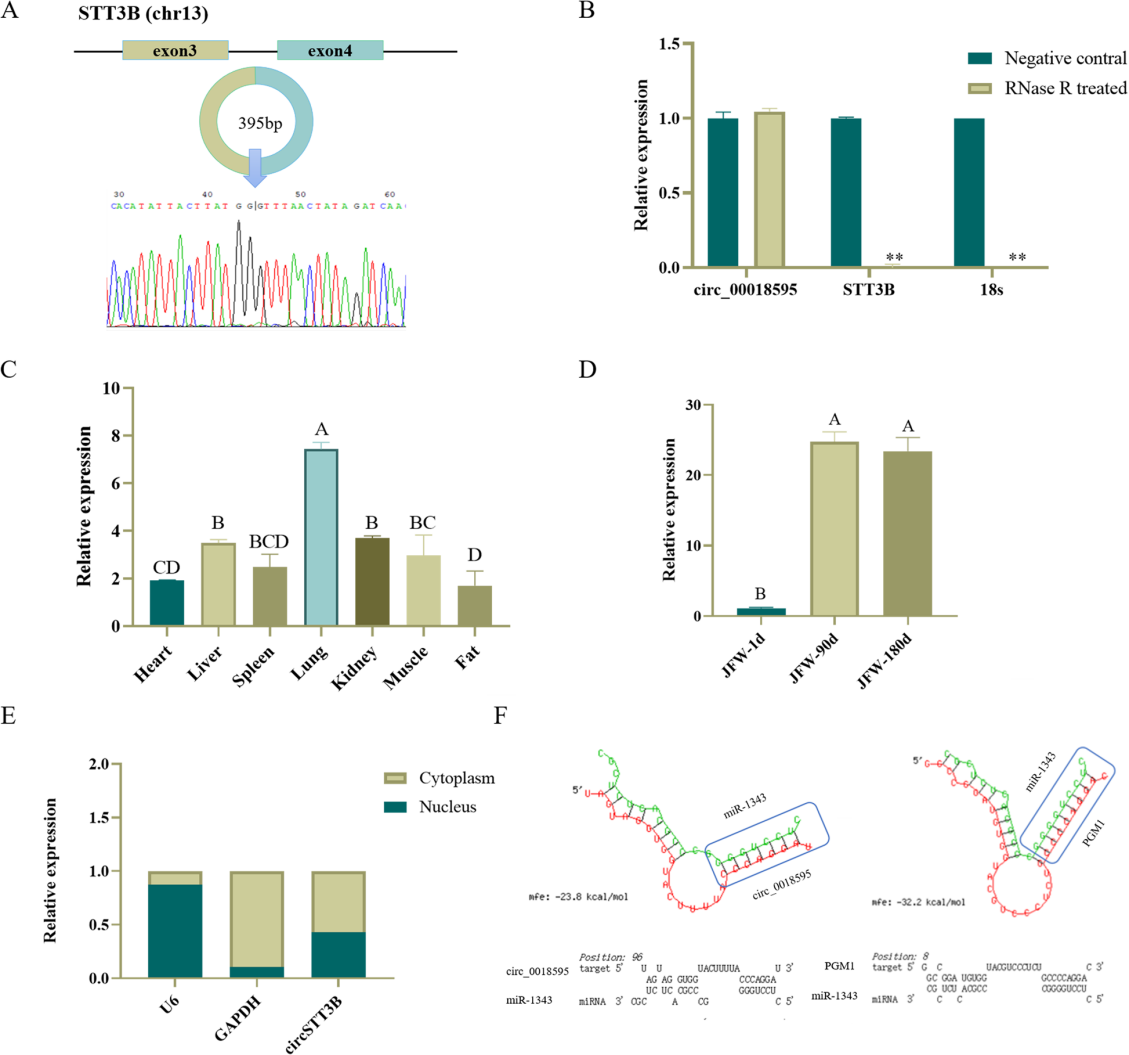
Characterization of circ_0018595. **A** Verification of circ_0018595 as a circRNA. **B** qRT-PCR results showing the resistance of circ_0018595 to RNase R digestion. **C** Expression levels of circ_0018595 in different tissues of Jinfen White pigs. **D** Expression changes of circ_0018595 in Jinfen White pigs at 1 d, 90 d, and 180 d. **E** Nuclear and cytoplasmic levels of circ_0018595 in porcine skeletal muscle satellite cells. **F** Modeled potential interactions between circ_0018595, *PGM1*, and miR-1343 generated using RNAhybrid. Note: In panels (**C**) and (**D**), uppercase letters are used to indicate extremely significant differences

From the above network, we selected circ_0018595 for follow-up study, speculating that it might be involved in regulating growth and development of porcine skeletal muscle via the predicted circ_0018595/miR-1343/*PGM1* axis (Fig. 5A). Circ_0018595 is an exonic circRNA generated from exons 3 and 4 of *STT3B*. It was amplified from longissimus dorsi cDNA using specific divergent primers, and the product was subjected to Sanger sequencing to validate the ligation site (Fig. 6A). We also processed the total RNA extract with RNase R and performed qRT-PCR, revealing that circ_0018595 was more resistant to RNase R than *STT3B* mRNA and 18S rRNA as expected (Fig. 6B). The circ_0018595 was expressed in various tissues, especially in lung, liver, skeletal muscle, and kidney (Fig. 6C). We also examined the expression of circ_0018595 in muscle of Jinfen White pigs at 1, 90, and 180 days, and found it was significantly associated with developmental stage, with strong upregulation between 1 and 90 days, and maintained at 180 days (Fig. 6D). Furthermore, the results of a nucleocytoplasmic separation assay showed that circ_0018595 is primarily localized in the cytoplasm, which was consistent with its proposed role as a sponge for miRNAs (Fig. 6E). Finally, the RNAhybrid website found that circ_0018595 and miR-1343 had potential binding sites, which was also found between miR-1343 and *PGM1* mRNA transcript (Fig. 6F). The function and mechanism experiment of circ_0018595 in the skeletal muscle development will be carried out in the future work.

## Discussion

CircRNAs are abundant in transcriptomes and participate in various biological activities by regulating transcription, splicing, mRNA stability, and translation. At present, many studies have investigated circRNAs as potential therapeutic targets for various diseases [45, 46]. With the ongoing development of related research strategies and methods, many biological functions of circRNAs in animals have also been emerging, and circRNAs have been found to regulate the proliferation and differentiation of animal skeletal muscle cells by acting as ceRNAs and encoding small peptides [47]. For example, Shen et al. [48], found that circTMTC1 can bind miR-128-3p and regulate expression of myostatin (*MSTN*), thereby inhibiting the proliferation and differentiation of chicken skeletal muscle satellite cells. Pigs are important farm animals that provide meat for humans and are often used for disease models in medical research. In 2017, Liang and coworkers constructed the first publicly available *Sus scrofa* circRNA database through analysis of nine organs of Guizhou pigs at three developmental stages [38]. Current research on circRNAs in porcine skeletal muscle mainly relies on the mining and discussion of sequencing data, and the specific mechanisms of circRNA involvement in skeletal muscle growth and fat deposition are rarely studied [49–51]. Here, we used RNA-seq to construct circRNA expression profiles for Jinfen White pig skeletal muscle at three developmental stages (1, 90, and 180 d). Compared with previous studies, our sequencing data are robust, with > 97% of bases meeting the Q20 threshold.

RNA-seq is widely used in the study of circRNA in model animals and non-model animals. We use the library construction method of linear RNA for RNA-seq identification. The circRNA detected by different software and algorithms is different. We use the intersection of circRNA predicted by find_circ and CIRC software can improve the accuracy of circRNA. This method has been used in mice [52], cattle [53], sheep [54], chicken [55] and other species [56]. We identified a total of 16,990 expressed circRNAs, which is much higher than the number reported in one recent study. We also analyzed the chromosomal distribution, composition, and length of these circRNAs. qRT-PCR confirmed the expression patterns derived from RNA-seq data for eight examples, further establishing the accuracy of our sequencing results, along with the developmental stage specificity of circRNA expression profiles. These findings are also consistent with previous studies [49–51]. Our results therefore enrich the known porcine circRNA library and confirm that circRNAs are consistent across pig species. In addition, we identified a total of 584 circRNAs that were differentially expressed between the three developmental stages. This may be helpful in identifying circRNAs that regulate skeletal muscle growth and development, and opening up new avenues of future exploration to elucidate the specific mechanisms involved.

Usually, circRNAs are generated by back-splicing of pre-mRNAs. They have notable structural stability and spatiotemporal specificity, and can positively or negatively regulate transcription of their parent genes [57]. We performed enrichment analysis on the host genes of all DE circRNAs identified in this study, and found that these host genes were mainly involved in the AMPK and cAMP signaling pathways. The AMPK signaling pathway is known as a regulator of oxidative metabolism in skeletal muscle, and it promotes the conversion of glycolytic muscle fibers into oxidative fibers [58, 59]. For example, in C2C12 myotubes, AMPK signaling drives expression of skeletal slow-twitch muscle fiber genes [60]. On the other hand, the cAMP signaling pathway affects protein synthesis rate, promotes skeletal muscle hypertrophy, and is expected to become a new target for the treatment of muscle mass loss during atrophic diseases [61–63]. Interestingly, more than one circRNA can be transcribed from a single gene due to alternative splicing mechanisms. In this study, we found that Lim-domain only protein 7 (*Lmo7*) produced 15 circRNAs, but among these, only circ_0016243 and circ_0016253 had high expression levels, suggesting that these two novel circRNAs may play important roles in cell differentiation in Jinfen White pigs. *LMO7* is essential for skeletal muscle development and encodes a transcription factor that binds to the *PAX3*, *MyoD*, and *Myf5* promoters to activate expression of key myogenic differentiation genes in C2C12 cells [64]. Thus, downregulation of *LMO7* was shown to inhibit myogenesis in chicken myoblasts [36]. In addition, circLMO7 (formed by circularization of exons 3–5 of bovine *LMO7*), can target miR-378a-3p to inhibit the differentiation and apoptosis of myoblasts, and the sequence of bovine circLMO7 is completely different from that of porcine circ_0016243 and circ_0016253 in our study [65].

As mentioned above, circRNAs can act as miRNA sponges to regulate the expression of target genes, through the ceRNA mechanism. We constructed a circRNA-miRNA-mRNA co-expression network using high-throughput sequencing of mRNA and miRNA expression network data. For some of the miRNAs in this ceRNA network, a role in regulating proliferation and differentiation of skeletal muscle satellite cells has already been reported in other animals. These include miR-370 [66], miR-214 [67], miR-133 [68], and miR-885 [69]. We predicted that circ_0000576 could act as a sponge for ssc-miR-15a, ssc-miR-98, and ssc-miR-885-3p, modulating the target genes of these miRNAs to regulate muscle growth and development. In addition, circ_0004213 is an exon-derived circRNA from the polypyrimidine tract binding protein 1 (*PTBP1*) gene, which itself encodes a splicing factor involved in regulatory processes related to cell differentiation, cell cycle and apoptosis, cell motility, cell metabolism, and the immune response [70]. We also predicted that circ_0004213 could target miR-370, which in turn targets α-actinin-3 (*ACTN3*). Involvement of miR-370 has been reported in many cellular functions, including cell proliferation, migration, and differentiation [66], while *ACTN3* encodes the sarcomeric α-actinin-3 protein that is a component of the Z line in mammalian skeletal muscle fibers [71]. We also identified circ_0018595, which is generated by cyclization of exons 3–4 of *STT3B* and is predicted to bind miR-1343. This miRNA is involved in cell proliferation and apoptotic processes, as well as affecting the pluripotency of stem cells. In the ovaries of highly fertile sows, miR-1343 suppresses expression of transforming growth factor-β receptor type 1 (*TGFBR1*) to inhibit pig granule cell proliferation and promote apoptosis, thus affecting sow fecundity [72]. Furthermore, miR-1343 specifically binds to the 3´UTR of Orthodenticle homeobox 2 (*OTX2)* and inhibits endogenous *OTX2* expression in pig pluripotent stem cells (piPSCs), enhancing the expression of pluripotency genes and thus maintaining piPSCs pluripotency in pigs [73]. Finally, we chose to focus specifically on *PGM1*, which has the highest expression level in porcine longissimus dorsi and biceps femoris. It is involved in the glycolytic pathway, which may be related to pork quality and productivity [74, 75], since reducing the proportion of glycolytic fibers in porcine longissimus dorsi can slow down the rate and extent of muscle pH decline after slaughter [76]. Thus, *PGM1* may be involved in regulating skeletal muscle growth and lipid deposition. Given these considerations, the circ_0018595/miR-1343/*PGM1* axis may play a key role in the growth and development of skeletal muscle in Jinfen White pigs.

We also have some limitations of this study. All circRNAs and their target miRNAs or mRNAs were predicted computationally, and the specific mechanisms of these circRNAs in regulating skeletal muscle still require experimental verification. A series of complex potential mechanisms, such as circRNAs interacting with proteins, regulating parental gene transcription, and participating in protein coding, which remain to be explored.

## Conclusion

This study identified and revealed the expression profile and potential role of circRNA in the longissimus dorsi muscle of Jinfen White pigs by using RNA-seq data. Based on enrichment analysis, the host genes of DE circRNA are mainly AMPK and cAMP pathway. In the constructed ceRNA network, a new axis in the network circ_0018595/miR-1343/*PGM*1 may participate in the regulation of skeletal muscle growth. In conclusion, we have identified a series of candidate circRNAs, which have potential regulatory effects on the growth and development of porcine skeletal muscle and provide important references for future research.

## Methods

### Experimental animals and samples

All experimental animals and procedures in this experiment were approved by the Institutional Animal Care and Use Committee of Shanxi Agricultural University (Shanxi, China). The 9 Jinfen White pigs used in the experiment came from Datong Pig Breeding Farm (Shanxi, China) and were raised under standard conditions without restriction on feeding and drinking. Three heads per group (denoted as samples JFW_1d_1, JFW_1d_2, JFW_1d_3; JFW_90d_1, JFW_90d_2, JFW_90d_3; JFW_180d_1, JFW_180d_2, JFW_180d_3) were sacrificed by corona and neck exsanguination on days 1, 90, and 180, respectively. Tissues such as heart, liver, spleen, lung, kidney, pancreas, longissimus dorsi and subcutaneous fat were quickly collected and immediately snap-frozen in liquid nitrogen, and stored at -80 °C for later use.

### Library preparation and RNA sequencing

Total RNA was isolated and purified using the Trizol reagent (Takara, Japan), following the manufacturer’s instructions. The concentration and purity of isolated RNA samples were tested by electrophoresis and further examined with a nucleic acid analysis system (Experion System, Bio-Rad, USA). The A_260_/A_280_ ratio was between 1.9 and 2.1, indicating that the extracted RNA samples were of good quality. Sequencing was done by Beijing Novogene, using the Illumina HiSeq 2500 sequencing platform for two-end (pair-end), high-throughput sequencing (2 × 150 bp), and the amount of sequencing data (clean reads) per library was about 10 Gb.

### Identification of differentially expressed circRNAs

To obtain high quality clean reads for subsequent bioinformatic analysis, raw data were filtered to remove low quality reads, adapter contamination, high unknown base content, and all rRNA-mapping reads. The Q20 and Q30 scores of the clean reads were calculated simultaneously, and circRNAs were then identified by aligning clean reads to the pig reference genome (Sscrofa 11.1) using find_circ and CIRI. The true expression levels of junction reads and circRNAs extracted from the sequencing data were affected by other factors, such as sequencing depth. Therefore, in order to accurately estimate true circRNA expression levels, the readcount data for predicted circRNAs were corrected using the TPM method. The filtered sequences were compared with the pig reference genome, the genomic features of each identified circRNA were analyzed, then the wider genomic and sequence characteristics of circRNAs in Jinfen White pigs were analyzed; mainly including the distribution among chromosomes, genomic location, and exon number of circRNAs.

### Gene Ontology (GO) and pathway enrichment analysis (KEGG)

Using GO analysis (http://www.geneontology.org/) and KEGG pathway enrichment analysis (https://www.kegg.jp/), the host genes of DE circRNAs at the three growth and developmental stages were categorized in order to understand their biological functions. In the GO analysis, using the pig reference genome as background, the host genes were mapped to different functional entries. Thereafter, the enrichment significance of each entry was expressed as the corrected q-value, where entries with *P* < 0.05 were considered to be significantly enriched. In the KEGG pathway enrichment analysis, the hypergeometric test was applied—again using the pig reference genome as the analysis background—to screen the significantly enriched pathways (*P* < 0.05) for the host genes, with the results plotted as a bubble chart.

### Target miRNA and gene prediction, and network analysis

To reveal functional interactions between ncRNAs and mRNAs, we used circRNA–miRNA interaction network analysis to construct a competing endogenous RNA network. Here, the public project TargetScan 7.0 (http://www.microrna.org/microrna/home.do) and miRanda software were used to predict potential regulatory relationships between circRNA, mRNA, and miRNA. Cytoscape (http://cytoscape.org/) was used for network visualization.

### Quantitative Reverse Transcription Polymerase Chain Reaction (qRT-PCR)

Total RNA was extracted from the nine muscle samples used in the RNA-seq experiments, then reverse transcribed using PrimeScript ® RT Master Mix (Takara, Japan) with divergent primers designed using Premier 3.0 (including qRT-PCR primers and PCR primer-specific alignment by NCBI Primer-BLAST). Details of primers used in this study are listed in Supplementary Table S3. The expression of each circRNA, as well as other parameters, was detected using a CFX96 quantitative PCR instrument (Bio-Rad). The relative expression of target circRNAs was calculated using the 2^−ΔΔCt^ method.

### Circularization site verification of circRNA

Eight selected circRNAs were amplified by PCR using the designed qRT-PCR primers and the longissimus dorsi cDNA as template. Then, 2% agarose gel electrophoresis and Sanger sequencing of the PCR products were used to confirm reverse splicing of each circRNA by identifying the junction sequence.

### RNase R enzyme digestion test

To test the circular nature of selected circRNAs, total RNA from porcine longissimus dorsi tissue was treated with RNase R enzyme (Epicentre) followed by qRT-PCR analysis.

### Statistical analysis

Data processing and production of graphics were carried out using SPSS and GraphPad Prism 6 software. To test for statistically significant differences between two averages, data were analyzed by Student’s t test, and *P* < 0.05 was considered to indicate statistical significance .

## Supplementary Information

**Additional file 1: Table S1.** The expression of all identified circRNAs.

**Additional file 2: Table S2.** CircRNA-miRNA-mRNA network.

**Additional file 3: Figure S1.** GO and KEGG results for 143 targeted mRNAs.

**Additional file 4: Table S3.** Primers used in this study.

## Data Availability

The circRNA, miRNA and mRNA raw sequence data reported in this paper have been uploaded to NCBI and are publicly accessible at PRJNA867525.

## Abbreviations

CircRNAs: Circular RNAs
DE: Differentially expressed
GO: Gene Ontology
KEGG: Kyoto Encyclopedia of Genes and Genomes
ceRNA: Competitive endogenous RNA
ncRNAs: Non-coding RNAs
miRNAs: MicroRNAs
lncRNAs: Long non-coding RNAs
pre-mRNA: Precursor mRNA
piPSCs: Pig pluripotent stem cells
MyoD1: Myoblast determination protein 1
Myf5: Myogenic factor 5
MyoG: Myogenin
Myf6: Myogenic factor 6
PAX3: Paired box 3
PAX7: Paired box 7
MEF2: Myocyte enhancer factor 2
TTLL7: Tubulin tyrosine ligase like 7
GADL1: Glutamate decarboxylase-like protein 1
MSTN: Myostatin
Lmo7: Lim-domain only protein 7
PTBP1: Polypyrimidine tract binding protein 1
ACTN3: α-Actinin-3
TGFBR1: Transforming growth factor-β receptor type 1
OTX2: Orthodenticle homeobox 2
PGM1: Phosphoglucomutase 1

## References

[1] Horak M, Novak J, Bienertova-Vasku J (2016). Muscle-specific microRNAs in skeletal muscle development. Dev Biol.

[2] Yin H, Price F, Rudnicki M (2013). Satellite cells and the muscle stem cell niche. Physiol Rev.

[3] Kuang S, Kuroda K, Le Grand F (2007). Asymmetric self-renewal and commitment of satellite stem cells in muscle. Cell.

[4] Verma N, Rettenmeier A, Schmitz-Spanke S (2011). Recent advances in the use of Sus scrofa (pig) as a model system for proteomic studies. Proteomics.

[5] Sanger H, Klotz G, Riesner D (1976). Viroids are single-stranded covalently closed circular RNA molecules existing as highly base-paired rod-like structures. Proc Natl Acad Sci USA.

[6] Brand-Saberi B (2005). Genetic and epigenetic control of skeletal muscle development. Ann Anat.

[7] Buckingham M, Relaix F (2007). The role of Pax genes in the development of tissues and organs: Pax3 and Pax7 regulate muscle progenitor cell functions. Annu Rev Cell Dev Biol.

[8] Sabourin L, Rudnicki M (2000). The molecular regulation of myogenesis. Clin Genet.

[9] Bi P, Ramirez-Martinez A, Li H (2017). Control of muscle formation by the fusogenic micropeptide myomixer. Science.

[10] Ito Y, Kayama T, Asahara H (2012). A systems approach and skeletal myogenesis. Comp Funct Genomics.

[11] Rudnicki M, Schnegelsberg P, Stead R (1993). MyoD or Myf-5 is required for the formation of skeletal muscle. Cell.

[12] Kassar-Duchossoy L, Gayraud-Morel B, Gomès D (2004). Mrf4 determines skeletal muscle identity in Myf5: Myod double-mutant mice. Nature.

[13] Hasty P, Bradley A, Morris J (1993). Muscle deficiency and neonatal death in mice with a targeted mutation in the myogenin gene. Nature.

[14] Myer A, Olson E, Klein W (2001). MyoD cannot compensate for the absence of myogenin during skeletal muscle differentiation in murine embryonic stem cells. Dev Biol.

[15] Li J, Zhao W, Li Q (2020). Long Non-Coding RNA H19 promotes porcine satellite cell differentiation by interacting with TDP43. Genes.

[16] Geng T, Liu Y, Xu Y (2018). H19 lncRNA promotes skeletal muscle insulin sensitivity in part by targeting AMPK. Diabetes.

[17] Raza S, Kaster N, Khan R (2020). The role of micrornas in muscle tissue development in beef cattle. Genes.

[18] Vicens Q, Westhof E (2014). Biogenesis of circular RNAs. Cell.

[19] Yuan X, Diao J, Du A (2020). Circular RNA expression profiles and features in NAFLD mice: a study using RNA-seq data. J Transl Med.

[20] Hao Z, Zhou H, Hickford J (2020). Identification and characterization of circular RNA in lactating mammary glands from two breeds of sheep with different milk production profiles using RNA-Seq. Genomics.

[21] Li M, Xie X, Zhou J (2017). Quantifying circular RNA expression from RNA-seq data using model-based framework. Bioinformatics.

[22] Li X, Yang L, Chen L (2018). The Biogenesis, functions, and challenges of circular rnas. Mol Cell.

[23] Memczak S, Jens M, Elefsinioti A (2013). Circular RNAs are a large class of animal RNAs with regulatory potency. Nature.

[24] Tao M, Zheng M, Xu Y (2021). CircRNAs and their regulatory roles in cancers. Mol Med.

[25] Shi X, Yang J, Liu M (2022). Circular RNA ANAPC7 inhibits tumor growth and muscle wasting via PHLPP2-AKT-TGF-β signaling axis in pancreatic cancer. Gastroenterology.

[26] Wang Y, Yan Q, Mo Y (2022). Splicing factor derived circular RNA circCAMSAP1 accelerates nasopharyngeal carcinoma tumorigenesis via a SERPINH1/c-Myc positive feedback loop. Mol Cancer.

[27] Fan H, Chen Z, Chen X (2022). METTL14-mediated mA modification of circORC5 suppresses gastric cancer progression by regulating miR-30c-2-3p/AKT1S1 axis. Mol Cancer.

[28] Ouyang H, Chen X, Wang Z (2018). Circular RNAs are abundant and dynamically expressed during embryonic muscle development in chickens. DNA Res.

[29] Chen X, Ouyang H, Wang Z (2018). A Novel Circular RNA generated by FGFR2 gene promotes myoblast proliferation and differentiation by sponging miR-133a-5p and miR-29b-1-5p. Cells.

[30] Yin H, Shen X, Zhao J (2020). Circular RNA CircFAM188B encodes a protein that regulates proliferation and differentiation of chicken skeletal muscle satellite cells. Front Cell Dev Biol.

[31] Li H, Yang J, Wei X (2018). CircFUT10 reduces proliferation and facilitates differentiation of myoblasts by sponging miR-133a. J Cell Physiol.

[32] Sun D, An J, Cui Z, et al. CircCSDE1 regulates proliferation and differentiation of c2c12 myoblasts by sponging miR-21–3p. Int J Mol Sci, 2022, 23(19):12038. 10.3390/ijms231912038 PMC9570022 36233353

[33] Ma L, Chen W, Li S (2022). Identification and functional prediction of circular RNAs related to growth traits and skeletal muscle development in duroc pigs. Front Genet.

[34] Yan J, Yang Y, Fan X (2022). CircRNAome profiling reveals circFgfr2 regulates myogenesis and muscle regeneration via a feedback loop. J Cachexia Sarcopenia Muscle.

[35] Cai B, Ma M, Zhou Z (2022). CircPTPN4 regulates myogenesis via the miR-499-3p/NAMPT axis. J Anim Sci Biotechnol.

[36] Shen X, Wei Y, Liu W (2021). A novel circular RNA circITSN2 targets the miR-218-5p/LMO7 axis to promote chicken embryonic myoblast proliferation and differentiation. Front Cell Dev Biol.

[37] Legnini I, Di Timoteo G, Rossi F (2017). Circ-ZNF609 is a circular rna that can be translated and functions in myogenesis. Mol Cell.

[38] Liang G, Yang Y, Niu G (2017). Genome-wide profiling of Sus scrofa circular RNAs across nine organs and three developmental stages. DNA Res.

[39] Kristensen L, Andersen M, Stagsted L (2019). The biogenesis, biology and characterization of circular RNAs. Nat Rev Genet.

[40] Das A, Shyamal S, Sinha T (2021). Identification of potential circRNA-microRNA-mRNA regulatory network in skeletal muscle. Front Mol Biosci.

[41] Zhang Y, Zhang X, Chen T (2013). Circular intronic long noncoding RNAs. Mol Cell.

[42] Li Z, Huang C, Bao C (2015). Exon-intron circular RNAs regulate transcription in the nucleus. Nat Struct Mol Biol.

[43] Kanehisa M, Furumichi M, Sato Y (2023). KEGG for taxonomy-based analysis of pathways and genomes. Nucleic Acids Res.

[44] Chen B, Yu J, Guo L (2019). Circular RNA circHIPK3 promotes the proliferation and differentiation of chicken myoblast cells by sponging miR-30a-3p. Cells.

[45] Cheng X, Ai K, Yi L (2022). The mmu_circRNA_37492/hsa_circ_0012138 function as potential ceRNA to attenuate obstructive renal fibrosis. Cell Death Dis.

[46] Deng W, Zhou X, Zhu K (2022). Novel circular RNA circ_0086722 drives tumor progression by regulating the miR-339-5p/STAT5A axis in prostate cancer. Cancer Lett.

[47] Wu X, Zhou J, Zhao L (2022). CircCYP24A1 hampered malignant phenotype of renal cancer carcinoma through modulating CMTM-4 expression via sponging miR-421. Cell Death Dis.

[48] Shen X, Liu Z, Cao X (2019). Circular RNA profiling identified an abundant circular RNA circTMTC1 that inhibits chicken skeletal muscle satellite cell differentiation by sponging miR-128-3p. Int J Biol Sci.

[49] Li B, Yang J, He J (2021). Spatiotemporal regulation and functional analysis of circular RNAs in skeletal muscle and subcutaneous fat during pig growth. Biology.

[50] Li M, Zhang N, Zhang W (2021). Comprehensive analysis of differentially expressed circRNAs and ceRNA regulatory network in porcine skeletal muscle. BMC Genomics.

[51] Qi K, Liu Y, Li C (2022). Construction of circRNA-related ceRNA networks in longissimus dorsi muscle of Queshan black and large white pigs. Mol Genet Genomics.

[52] Jakobi T, Czaja-Hasse L, Reinhardt R (2016). Profiling and validation of the circular RNA repertoire in adult murine hearts. Genomics Proteomics Bioinformatics.

[53] Yang Xinran, Ma X, Mei C, et al. A genome-wide landscape of mRNAs, lncRNAs, circRNAs and miRNAs during intramuscular adipogenesis in cattle. BMC Genomics, 2022, 23: 691. 10.1186/s12864-022-08911-z PMC9535873 36203142

[54] Wang J, Chen H, Zhang Y, et al. Comprehensive analysis of differentially expressed circRNAs in the ovaries of low- and high-fertility sheep. Animals, 2023, 13:236. 10.3390/ani13020236 PMC9854751 36670776

[55] Zhang G, Zhang J, Wu P (2022). Transcriptome sequencing analysis of circRNA in skeletal muscle between fast- and slow-growing chickens at embryonic stages. Animals.

[56] Chen X, Wang Z, Chen Y (2022). Transcriptome analysis of differentially expressed circRNAs miRNAs and mRNAs during the challenge of coccidiosis. Front Immunol.

[57] Barrett SP, Salzman J (2016). Circular RNAs: analysis, expression and potential functions. Development.

[58] Witczak C, Sharoff C, Goodyear L (2008). AMP-activated protein kinase in skeletal muscle: from structure and localization to its role as a master regulator of cellular metabolism. Cell Mol Life Sci.

[59] Lee Y, Yun M, Kim H (2016). Exogenous administration of DLK1 ameliorates hepatic steatosis and regulates gluconeogenesis via activation of AMPK. Int J Obesity.

[60] Xu M, Chen X, Huang Z (2020). Procyanidin B2 promotes skeletal slow-twitch myofiber gene expression through the ampk signaling pathway in C2C12 myotubes. J Agric Food Chem.

[61] Lauterbach N, Gonçalves D, Silveira W (2022). Urocortin 2 promotes hypertrophy and enhances skeletal muscle function through cAMP and insulin/IGF-1 signaling pathways. Molecular Metabolism.

[62] Machado J, Manfredi L, Silveira W (2016). Calcitonin gene-related peptide inhibits autophagic-lysosomal proteolysis through cAMP/PKA signaling in rat skeletal muscles. Int J Biochem Cell Biol.

[63] Tong T, Kim M, Park T (2019). α-Ionone attenuates high-fat diet-induced skeletal muscle wasting in mice via activation of cAMP signaling. Food Function.

[64] Dedeic Z, Cetera M, Cohen T (2011). Emerin inhibits Lmo7 binding to the Pax3 and MyoD promoters and expression of myoblast proliferation genes. J Cell Sci.

[65] Wei X, Li H, Yang J (2017). Circular RNA profiling reveals an abundant circLMO7 that regulates myoblasts differentiation and survival by sponging miR-378a-3p. Cell Death Dis.

[66] Zhang P, Du J, Guo X (2021). LncMyoD promotes skeletal myogenesis and regulates skeletal muscle fiber-type composition by sponging miR-370-3p. Genes.

[67] Du Y, Wang Y, Li Y, et al. MiR-214-5p regulating differentiation of intramuscular preadipocytes in goats via targeting KLF12. Front Genet. 2021;12:748629. 10.3389/fgene.2021.748629 PMC8730364 35003206

[68] Feng Y, Niu L, Wei W (2013). A feedback circuit between miR-133 and the ERK1/2 pathway involving an exquisite mechanism for regulating myoblast proliferation and differentiation. Cell Death Dis.

[69] Elsaeid Elnour I, Dong D, Wang X (2020). Bta-miR-885 promotes proliferation and inhibits differentiation of myoblasts by targeting MyoD1. J Cell Physiol.

[70] Zhu W, Zhou B, Rong L (2020). Roles of PTBP1 in alternative splicing, glycolysis, and oncogenesis. J Zhejiang Univ Sci B.

[71] Harada N, Gotoda Y, Hatakeyama A (2020). Differential regulation of Actn2 and Actn3 expression during unfolded protein response in C2C12 myotubes. J Muscle Res Cell Motility.

[72] Hu H, Fu Y, Zhou B, et al. Long non-coding RNA TCONS_00814106 regulates porcine granulosa cell proliferation and apoptosis by sponging miR-1343. Mol Cell Endocrinol. 2021;520:111064. 10.1016/j.mce.2020.111064 33091558

[73] Xie Y, Cao H, Zhang Z (2019). Molecular network of miR-1343 regulates the pluripotency of porcine pluripotent stem cells via repressing OTX2 expression. RNA Biol.

[74] Yu J, Shao S, Xiong Y (2015). Molecular characterization, expression patterns, and promoter activity analysis of PGM1 in pigs. Genet Mol Res.

[75] Xing K, Wang K, Ao H (2019). Comparative adipose transcriptome analysis digs out genes related to fat deposition in two pig breeds. Sci Rep.

[76] Kim G, Jeong J, Yang H (2019). Differential abundance of proteome associated with intramuscular variation of meat quality in porcine longissimus thoracis et lumborum muscle. Meat Sci.

