# Supplementary figures and images for: Genome-wide analysis of circular RNA-mediated ceRNA regulation in porcine skeletal muscle development
Source: BMC Genomics. 2023 Apr 12;24:196. doi: 10.1186/s12864-023-09284-7 (PMC10099641; doi:10.1186/s12864-023-09284-7)

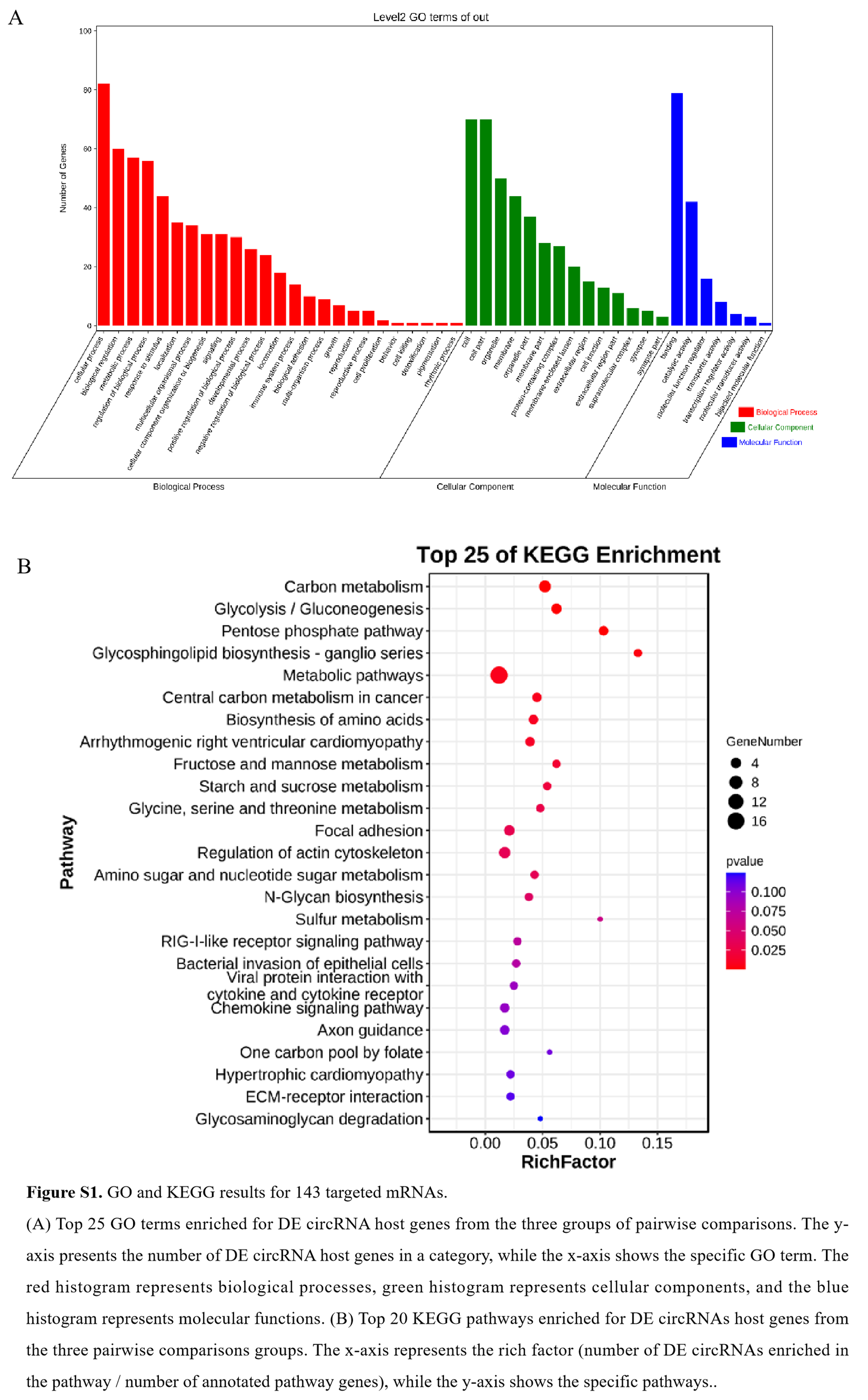

Supplement: Supplementary file 3 — Additional file 3: Figure S1. GO and KEGG results for 143 targeted mRNAs. [file 12864_2023_9284_MOESM3_ESM.png]
