# Supplementary material for: Genome-wide analysis of circular RNA-mediated ceRNA regulation in porcine skeletal muscle development
Source: BMC Genomics. 2023 Apr 12;24:196. doi: 10.1186/s12864-023-09284-7 (PMC10099641; doi:10.1186/s12864-023-09284-7)
Supplement: Supplementary file 4 — Additional file 4: Table S3. Primers used in this study. [file 12864_2023_9284_MOESM4_ESM.docx]

**Table S3 Primers used in this study**

| **Name** | **Primer sequence (5’→3’)** | | **Tm (℃)** | **Product Size (bp)** |
| --- | --- | --- | --- | --- |
| circ_0018595  circ_0018595 | convergent primer | F: TGTGTGTTCCTTGCACCAAC  R: AAGCAGCTAAAAGTCCCGCT | 60 | 80 |
|  | divergent primer | F: GCGGGACTTTTAGCTGCTTG  R: TGAGACGCAAGATGATGTGTTG | 59 | 159 |
| circ_0009450 | divergent primer | F: CGTTCTGTTGCCACTCTCCT  R: ATGGCCTGTCAGTTGATGTTCT | 60 | 100 |
| circ_0010243 | divergent primer | F: TTGGCTACTTCGTGCAGCTA  R: TCCTTATGTGACTGTCGGCTG | 60 | 143 |
| circ_0025192 | divergent primer | F: TTCTTCGGCAGCTTACCCAC  R: ATATGGGCTTCTGCTTCGGG | 60 | 124 |
| circ_0013402 | divergent primer | F: TTAGAGCAGGGCTATGCAGC  R: TTTCACAACTTGGCAAGAGC | 59 | 154 |
| circ_0019993 | divergent primer | F: AGGGAATCCCAATCACCCCT  R: AGCCCACGGTATTCTCGTTT | 60 | 140 |
| circ_0017860 | divergent primer | F: TTTGTCCAGTGGAAGACCACC  R: GTTGCTGCATTCGAGAAGAGG | 60 | 195 |
| circ_0016243 | divergent primer | F: ACACAAAACGGTGGGAGGAA  R: TGATTGGGCTTAGCTTTCGC | 59 | 239 |
| STT3B |  | F: TGGAGGAGGCCTTTACCTCA  R: CTCGCTTCCTTTTTGCGGTC | 60 | 153 |
| U6 |  | F: CTCGCTTCGGCAGCACA  R: AACGCTTCACGAATTTGCGT | 60 | 94 |
| CAPDH |  | F: TCGGAGTGAACGGATTTGGC  R: TGACAAGCTTCCCGTTCTCC | 60 | 189 |
| *18S rRNA* |  | F: CCCACGGAATCGAGAAAGAG  R: TTGACGGAAGGGCACCA | 60 | 132 |
